# Neonatal Weight and Prenatal Exposure to Polychlorinated Biphenyls: A Meta-Analysis

**DOI:** 10.31557/APJCP.2019.20.11.3251

**Published:** 2019

**Authors:** Hongling Zou, Yinxia Lin, Liu Yang, Chaoyan Ou, Fang Geng, Yao Wang, Wanrong Chen, Yali Niu, Rimei Liang, Qianzi Su, Yi Sun

**Affiliations:** 1 *Department of Oncology, Second Affiliated Hospital of Guilin Medical College, *; 2 *Department of Toxicology, Guilin Medical University School of Public Health, Guilin, *; 3 *Department of Clinical Laboratory, Tangshan Maternity and Children’s Health Care Hospital, Tangshan, Hebei, China. *

**Keywords:** Polychlorinated biphenyls, birth weight, meta-analysis

## Abstract

**Background:**

This research studied the relationship between maternal exposure to polychlorinated biphenyls and neonatal birth weight through systematic review and meta-analysis of existing literature.

**Methods:**

We searched for all the studies published in MEDLINE / PUBMEDN / EMBASE (Medical Abstract Database) by June 2018, and seven studies had been selected.

**Results:**

The results showed that there was significant correlation between birth weight reduction and PCBS exposure throughout pregnancy (β=-0.586g, 95%CI:-0.629,-0.543). There was a negative correlation between birth weight and PCBs exposure and umbilical cord serum (β=-0.833g) and maternal serum (β= -0.504g).Subgroup analyses showed significantly different effects of PCBs exposure on birth weight in different regions, stages of pregnancy and study designs. It was thought the heterogeneity was mainly caused by geographical regions, stages of pregnancy, and the assessment methods.

**Conclusion:**

The meta analysis revealed a negative correlation between PCBs exposure and birth weight but there was significant difference in the correlation between birth weight loss.

## Introduction

Polychlorinated biphenyls (PCBs) are lipophilic and poorly metabolized compounds, which tend to be accumulated in adipose tissue of aquatic organisms in polluted water. These compounds may have many adverse effects on infant development through placenta and breast feeding (Kanae et al., 2009) Birth weight is an important indicator of fetal growth, and Low birth weight (LBW) is associated with perinatal morbidity and mortality. Moreover it was also found that the adolescent and middle-aged health issues including asthma, low IQ, and hypertension could be caused by LBW or premature birth (Harris et al., 2014). Therefore, it was important to find the risk factors which caused LBW,and it was significant to reduce the LBW incidence for public health. 

In the past, more and more studies had estimated the association of PCBs exposure with birth weight and LBW. Many studies had reported inconsistent findings regarding the relationship between maternal PCBs exposure and birth weight. Some studies have suggested a negative correlation between PCBs and birth weight in newborns (Fein et al.,1984; Karmaus et al., 2004; Murphy et al., 2010; Patandin et al., 1998; Rylander et al,1985; Rylander et al.,1996; Rylander et al., 1998; Wojtyniak et al.,1998), while others had shown that the relationship between PCBs and birth weight was not clear (Gladen et al., 2003; Grandjean et al., 2001; Longnecker et al., 2005; Mendez et al., 2010; Vartiainen et al., 1998). These inconsistent and controversial results suggested there was an urgent need to quantitatively synthesize and explain the evidence available in order to promote public health policy and ensure informed clinical decisions. PCBs were lipophilic compounds and contain different amounts of polychlorinated biphenyls in different tissues. So there was a lot of controversy when referred to the different sample extraction. And no previous research has shown the effect of PCBs in different tissues on birth weight. Therefore, we carried out meta- analysis to quantitatively synthesize the results of various studies. 

In this analysis, we systematically collected previously published studies to estimate the effects of PCBs exposure in different tissues on birth weight and/or LBW. Meta-analysis model was used to quantitatively evaluate the effect of PCBs exposure and the exposure evaluation method on birth weight and LBW. Finally we further discussed the effects of different regions, research settings and research designs in subgroup analysis. 

## Materials and Methods

*Study design*

We searched all studies published in MEDLINE, PubMed, Embase, China Biomedical and Wanfang Electronic Databases before June 2018. Our search strategy combined the following keywords: Biphenyls, Polychlorinated, Polychlorinated biphenyls Compounds, Polychlorinated biphenyls PCBs, Birth Weight Synonyms, Low Birth Weights, Infants Weight, change in birth weight, LBW, adverse birth outcomes, and adverse pregnancy outcomes. We also manually searched for additional publications for each major research reference. Further publications are also determined through reviewing articles, but only in English or Chinese publications.

*Inclusion and exclusion criteria*

Firstly we screened the abstract of all research articles to exclude studies which were not involve in the relationship between PCBs and birth weight. The remaining studies were labeled as potential and further evaluated by two independent authors. Studies which met the following criteria were included into the meta analysis: (a) Studies included PCBs exposures during pregnancy, and pregnancy outcomes measured birth weight and/or LBW; birth weight was measured as a continuous variable; LBW was defined as live births with a weight of less than 2500g, including terminology LBW (TLBW) and preterm LBW(PLBW); (b)The study provided LBW birth weight, sample size, partial regression coefficient (β) odds ratio (OR), and 95% confidence intervals (CI), which could also be used to infer the results of the study; (c) If more than one study was indentified for the same population, only student that included the latest population or the most information were selected. Accordingly, studies those didn’t conform to the above criteria were excluded. 

*Data Extraction*

The following information was extracted from each study: year and source of publication, study period, study setting, study plan, PCBs exposure detection method, data source, sample size, PCBs exposure window, exposure mean and range,β/OR and 95% CI. If the study provided an association between PCBs exposure throughout pregnancy and birth weight during specific periods of pregnancy, all estimates were extracted. Some studies assessed PCBs exposures based on monitoring network data and remote sensing data, and the estimates based on the monitoring network were preferred, because it was more commonly used and potentially reduced the heterogeneity among studies in the meta-analysis. In addition, if other covariances were fully adjusted, estimates could only be extracted from a single contaminant model because there was a considerable collinear relationship in the model of the contaminants. Sometimes there was considerable collinearity in the pollutants from the same source, as a result, estimates were extracted from a single pollutant model only when covariance had been fully adjusted. If the pollutant didn’t exist alone, there would be combined effect. However, it wasn’t every studies adjusted those contaminants except PCBs. Qualification assessments and all data extraction in standard forms were performed by two authors, and differences were resolved after discussion.

*Meta-analysis and Statistical Analysis*

Before meta-analysis were carried on, we converted all risk estimates (β and OR) of PCBs volume and chemical composition to common exposure unit, which allowed us to pool the estimates from different studies. To compare exposure level in cohort results with different matrices, we showed the contaminants levels in wet-weight umbilical cord serum levels, which directly reflected fetal exposure during childbirth (some compounds do not effectively cross the placenta). If there were no umbilical cord blood results, we estimated the concentrations in maternal serum, maternal whole blood, and breast milk. And the following conversion factors were used (Eva Govarts, et al. ,2012).

Level of umbilical cord serum (ng/L) = 0.20 × level of pregnancy serum (ng/L) = 1.20 ×level of breast milk (ng/g fat) = 0.36 x level of materials’ whole blood (ng/L).

Moreover, we conducted several meta-analysis studies to quantitatively estimate the relationship between different PCBs concentrations and birth weight. Minor analyses were also performed to estimate the summary effects of PCBs exposure on birth weight throughout pregnancy in the subgroups with different exposure measurement methods , study design and study settings. These subgroup analyses were designed to investigate the what important role these characteristics play in the effect of PCBs exposure on birth weight ,and to further detected whether the characteristics took effect on the heterogeneity in the reported association. The studies included four detection methods to evaluate PCBs levels ,including umbilical cord serum, maternal serum, maternal serum and breast milk. Cord blood serum was collected from cord blood samples at the time of birth, and the serum fraction was removed after centrifugation and stored at -20^o^C. The levels of PCBs in umbilical cord serum is a good indication of the infant exposures to PCBs, and the results were reproducible (Sharon et al., 2007). PCBs levels in pregnancy serum were detected in blood samples taken from pregnant women during the first trimester, the second trimester, or the third trimester of pregnancy respectively (Lars et al., 2000; Matthew et al., 2005; Halldorsson et al., 2008; Marina et al, 2014). However, thePCBs levels in pregnant women appeared to be relatively stable throughout pregnancy. All studies included in the study were divided into two categories: retrospective and prospective. Meta-regression analyses were also used to explore the revision effect of the study period, exposure measurement methods, study design and study settings on the association between PBCs exposure and birth weight.

To investigate the heterogeneity of the results, we assumed that the effect magnitude may changed with the study method. The inclusion heterogeneity of the studies was assessed via the Q and I² statistics. Cochran’s Q statistic was calculated by the square of the summation, and weight were conducted with each study’s contribution , each study’s estimate and the deviation of overall meta-estimation P values were obtained by comparing Q statistic with a chi-square distributionwith k-1 degrees of freedom, where k was the number of studies included (Higgins et al., 2003). If the P-value was less than 0.05, then random effects model was selected on the other side the fixed effects model was selected. The I² statistic [I² = (Q-df) / Q × 100] described the percentage of inter-study variation caused by heterogeneity rather than occasionality. If I²>50%, it indicated that there was significant statistical heterogeneity . We also used the asymmetry of funnel plot to detect potential publication bias. The symmetry of funnel diagramwas tested with Egger regression, where the reciprocal of the standard error was an independent variable and the standardized estimate of effect size was the dependent variable (Egger et al., 1997).

Finally, a series of sensitivity analysis were conducted to test the robustness of our results. Because some subgroup analyses contained few studies, only sensitivity analysis were performed in those subgroup with more than three studies. For each sensitivity analysis, we removed the largest and the smallest estimates from a single study separately from the meta-analysis. All statistical tests were bilateral, and P <0.05 was considered statistically significant. STATA software and “mate for” package were used in the analysis.

## Results

*Search results and research features*

**Figure 1:**
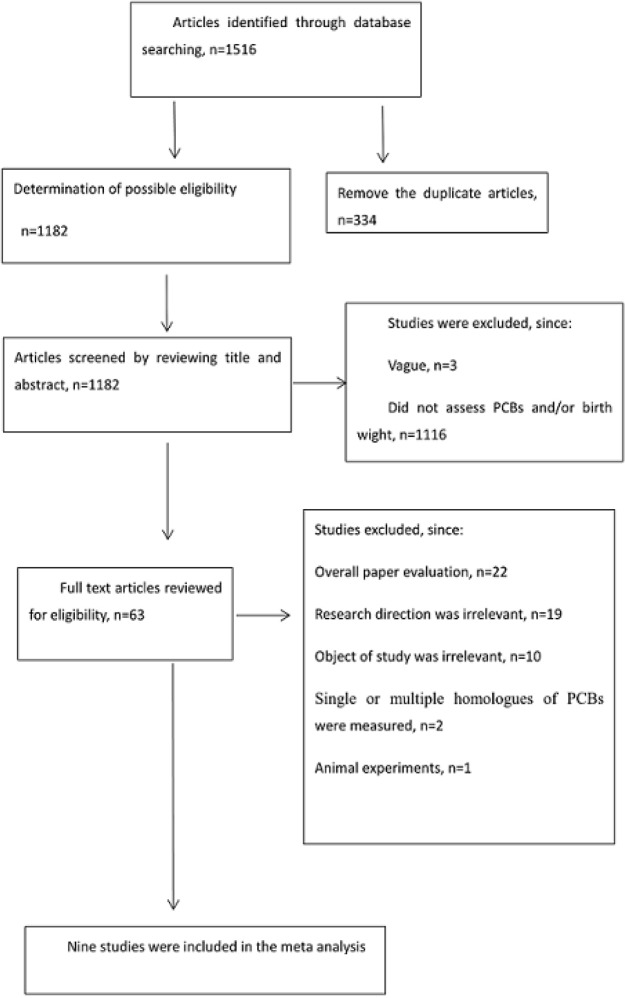
Flow Chart of the Selection Process

The search strategy identified 1,516 articles, and 334 articles were duplicates among those. The titles and the abstracts of the remaining 1,182 studies were reviewed, and 1,119 studies (The contents of the 3 articles were vague and can not determine the specific research substance, 1,116 articles did not assess PCBs and/or birth weight) were excluded. The full text of 63 studies were reviewed and 56 studies were further excluded, because 22 articles are mainly analysis and summary previous studies or lack of specific data and he research direction of 19 studies was irrelevant, the research objects of 10 articles were irrelevant, single or multiple homologues of PCBs were measured in 2 articles, 2 articles was published a long time ago and 1 articles was animal experiments. Finally, 7 studies was included in the meta-analysis, including Matthew et al., 2005; Xijin et al., 2015; Marina et al, 2014; Lars et al., 2000; Kanae et al., 2009; Halldorsson et al., 2008; Sharon et al., 2007). The selection process was shown in Figure 1, and the characteristics of the eligible studies were shown in Table 1.

*Comprehensive estimation of the effect of PCBs exposure on birth weight*

It is estimated that there is a significant negative correlation between birth weight loss(β=-0.586g, 95%CI: -0.629, -0.543) and PCBs exposure, as shown in tables 2 and 2. The combined effect of the two studies from Asia was(β=-0.396g, 95%CI: -0.519, -0.272), P > 0.05, indicating that the combined effects of the two studies were not statistically significant (Figure 3B). Most of the combined effects in other groups were statistically significant (Figure 3). For two studies using cord blood serum as a sample, the level of PCBs in cord serum was 1.7 μ g / L, the average birth weight loss was 9.52 g, and the combined effect was (β=-0.833g, 95%CI: -1.695,-0.029) (Figure 3A). In six late studies, the combined effect values were (β=-0.657g, 95% CI / 0. 905,-0.410), one of which was OR = 0. 3852, indicating that the concentration of PCBs in maternal serum was 1. 2564 μ g / L. PCBs is not a risk factor for loss of birth weight, but it can lead to premature delivery, while another study had an OR value of 1.6, but no concentration of PCBs, indicating that pregnant women were exposed to a certain level of PCBs, in the third trimester of gestation, the risk of birth weight loss is high (Figure 3C).

There was significant heterogeneity in these 7 studies (P < 0. 853). In order to explore the source of heterogeneity in the study, a series of subgroup analysis was carried out. The results show that race is the biggest cause of heterogeneity. In particular, the largest factor contributing to the study of heterogeneity in Europe (P=0.839) (Figure 3B). Three studies from Europe using maternal serum as an exposure assessment item were included in the meta analysis with their combined value (β=-0.601g, 95% CI: -0.650, -0.551). In addition, late pregnancy in pregnancy staging (β=-0.657g, 95% CI: -0.905, -0.410)(Figure 3C), maternal serum in exposure test samples(β=-0.504g, 95% CI:-0.785,-0.223)(Figure 3A), prospective research in research design (β=-0.631g, 95% CI:-0.910, -0.351)(Figure 3D), are the cause of heterogeneity.

*Sensitivity Analysis and Publication Bias Analysis*

Finally, we conducted a series of sensitivity analyses to determined the robustness of the results. An American study was excluded in our meta-analysis which detected the PBCs level in cord blood and had maximum impact, and we found no change (β=-0.486g, 95%CI:-0.737,-0.234). The robustness of a single study with the maximum and minimum impact on estimating the relevance of correlation. There was statistically significant (β=-0.631g, 95%CI:-0.910,-0.351) in estimated aggregate effects of birth weight (β= -0.319g), as shown in Figure 4 and 6. The results showed that neither Begg’s test (P = 0.652) nor Egger’ s test (t=0.22 P = 0.835) found obvious publication bias in the included articles. The results of Egger showed that meta-analysis had no significant publishion bias, as shown in Tables 2 and Figure 5.

**Table 1:** Characteristics of the Studies in the Meta-Analysis

Measuring items	Race	Country	Authors	Research persistence time	Partake quantity	OR/β(95%CI)	Average potency	Chemical composition	Case type
Pregnant women serum	America	United States	Matthew et al., 2005	1938-1965	1,034	OR=0.3852 (0.2304, 0.6372)	1.2564μg /L	—	premature birth
Umbilical cord serum	Asia	China	Xijin Xu et al., 2015	2010-2011	282	—	—	PCB homolog 28,52,101,138,153,180,209	infant of low-birth weight
Pregnant women serum	Europe	Greece	Marina et al., 2014	2007-2014	1,117	β=−0.03132 (−0.05976−0.002844)	1.8μg/L	PCB118,PCB138,PCB153,PCB156,PCB170,PCB180	infant of low-birth weight
Maternal serum	Europe	Sweden	Lars et al., 2000	1973-1993	4,401	OR=1.6 (1.1-2.3)	—	—	infant of low-birth weight
Maternal serum	Asia	Japan	Kanae et al., 2009	2002-2005	398	β=−125.9	—	—	infant of low-birth weight
Maternal serum	Europe	Denmark	T. I. et al., 2008	1996-2002	100	β=0.6	0.23μg/L	—	infant of low-birth weight
Umbilical cord serum	America	United States	Sharon et al 2007	1993-1998	722	β=−9.52	1.7μg/L	—	infant of low-birth weight

Note, means data deficiency

**Figure 2:**
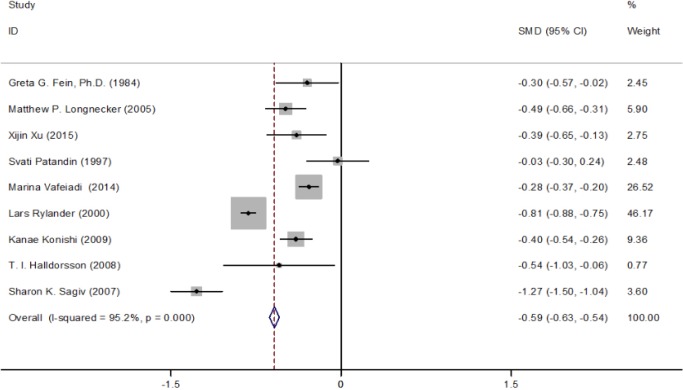
Forest Plots for the Association between PCBs Exposure (Per 1μg/l Increments) and Birth Weight (β,95%CI)

**Table 2:** Joint Association between Pcbs Exposure During Pregnancy and Changes in Birth Weight in Different Subgroups

Subunit	Number of studies	The p of Heterogeneity Test	β (95% CI)	I^2^(%)	P of Egger test
Exposure throughout pregnancy	7	0	-0.598 (-0.852,-0.343)	95.90	0.853
Given period					
First March exposure	3	0.084	-0.386 ( -0.559, -0.213)	59.70	0.39
Second exposures in March	2	0.0828	-0.494 ( -0.660, -0.328)	0	-
Third March exposure	6	0	-0.657 ( -0.905, -0.410)	92.30	0.337
Race					
Asia	2	0.969	-0.396 (-0.519, -0.272)	0.00	-
Europe	3	0	-0.601 (-0.650, -0.551)	98.00	0.839
America	2	0	-0.876 (-1.644,-0.108)	96.50	-
Exposure assessment items					
Cord serum	2	0	-0.833 (-1.695,-0.029)	96	-
Maternal serum	5	0	-0.504 (-0.785,-0.223)	96.30	0.561
Research design					
Perspective study	6	0	-0.631 (-0.910, -0.351)	96.50	0.38
Retrospective study	1	-	-0.319 (-0.650, -0.132)	-	-

**Figure 3:**
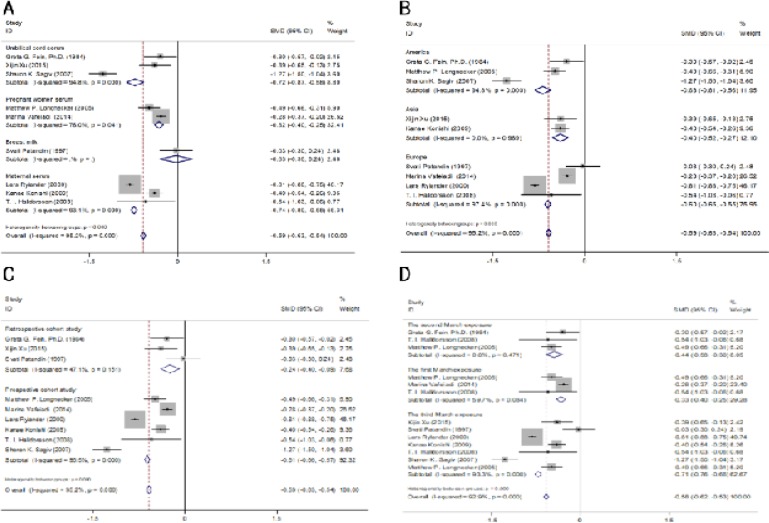
Forest Plots for the Association between PCBs Exposure (per 1μg/l Increments) and Birth Weight (β,95%CI) in Different Subgroups

**Figure 4:**
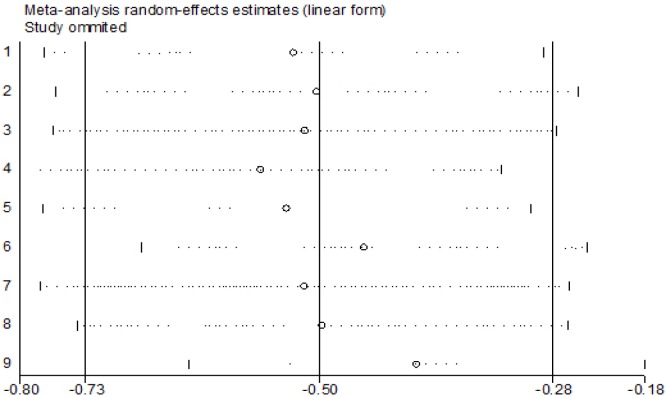
Sensitivity Analysis: Excluding the smallest study, the correlation between the estimated correlation effects of birth weight

**Figure 5:**
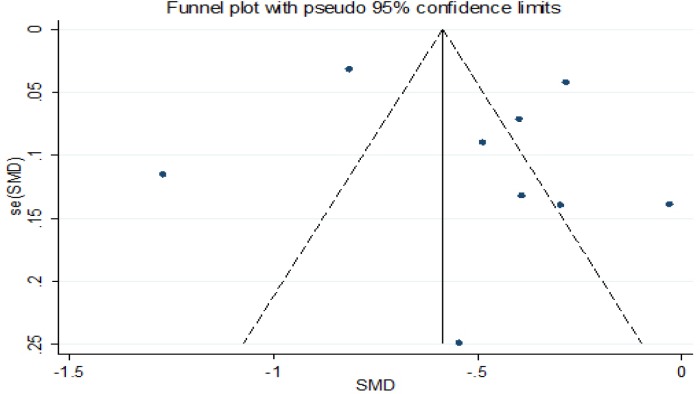
Funnel Diagram:Relationship between polychlorinated biphenyls (β,95%Cl)and birth weight(g).

**Figure 6:**
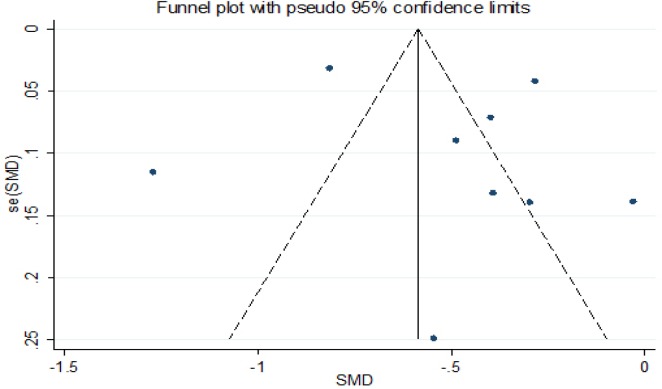
Funnel Diagram

## Discussion

In this meta-analysis, we collected seven eligible studies and quantitatively assessed the relationship between PCBs mass concentration and birth weight and/or LBW risk, involving a total of 8,054 subjects. The results showed that the exposure of PCBs was negatively correlated with the risk of low birth weight but there was significant difference in the correlation between birth weight loss (β= -0.586g, 95% CI: -0.629,-0.543) and PCBs exposure during pregnancy (P < 0.001). It was consistent with previous meta analysis results (Nina et al., 2015; Marc-André et al., 2013). Karmauspointed out that the birth weight reduced about 500g if PCBs level in mother was more than 25μg/L (Karmaus et al.,2004). Eva’s meta analysis found, the birth weight loss was 150g (95% CI: -250 -50 g) as 1 μ g/L PCB-153 increasing (Eva Govarts, et al.,2012). Nina’s meta analysis found that when PCB-153 concentration was beyond 183ng/g, newborn birth weight loss was about 140g (Nina et al., 2015). These results further revealed the toxicity of PCBs to fetal growth during pregnancy. Nowadays, PBCs was found in all of the world, and most pregnant woman faced exposed to PBCs more and less, as a result, these fetuses who were more susceptible to pollutants and were in a critical development stage, could be most risk situation. Therefore, it was critical to eliminate environmental PCB exposure to pregnant womanin in order to improve fetal health.

It was not sure the relative fators which effect the toxic mechanism of PCBs until now. Previous studies found that male infants were more susceptible to PCBs than female infants. However, there was few strong experimental evidences to prove this phenomenon. During the literature screening process, we found that the effects of PCBs, dioxin, polychlorinated biphenyls (PCDF) on birth weight in most studies, but there was a few studies to explore the combined effects of these pollutant with PCBs. Therefore, it will pay more attention to joint exposure of PBCs, the genes relative to PBCs in humans, or environmental gene interaction between gene an PBCs.

It was still controversial which was the most right and sensitive tissue to detect pops pollution. It is well known that human body were composed with all kinds of substances and the distribution of substances in human body were inconsistent. Therefore it is reasonable to conduct a meta-analysis to evaluat the analytical differences of PCBs from different tissue. In this study, we divided all of the studies into two groups (cord blood , maternal serum). For the analysis of, we observed that the there was more significant correlation between birth weight and PCBs levels in cord blood than in the blood of pregnant women. Our study also found that the birth weight was different at different concentration of PCB in cord blood while the PCB concentration of maternal serum were almost similar in prenatal (Sharon et al., 2007). This difference may has relationship with the oganizational distribution of PCBs in vivo. However, PCB were detected in placental tissue in some studies (Xu et al., 2015; Matthew et al., 2005), and we believe that new method will appear in other tissue in the future. In addition, studies have shown that the serum concentrations of Polychlorinated biphenyls congeners at 6 weeks after delivery and in early pregnancy were significantly lower than those before conception, so that different exposure items were detected at the same time. The content of PCBs was also detected at different periods of time.

Several subgroup analyses were performed to determine the heterogeneity sources in the studies. Heterogeneity test showed that the most likely source of heterogeneity between PCBs exposure and birth weight was different ethnic groups. The toxicity of PCBs to humans may vary with race. There could be some other factors, such as gene, heredity, individual differences and so on. Different food habits may also be the cause of this difference. The eating habits in different countries and regions was one of the reason that cause different PCBs level in human. For example, the femal subjects were divided into subgroups depended on fish consumption in some studies.It was found a correlation between fish intake and PCB concentration in body (Halldorsson et al., 2008). And Xijin et al.,’s (2015) study also showed that the similar effects of dietary intake on PCBs level. In addition, different standards and methods for the PCBs detection in various countries were different , which may have an impact on the results of the study. 

In the subgroup analysis, our results showed that there was a stronger aggregate association between PCBs exposure and LBW in late pregnancy than in the evaluation of PCBs exposure in the second and early stages of pregnancy. In Marc-Andr é et al.,’s (2013) study, the link between PCBs levels and birth weight may be due to weight gain during pregnancy. However, studies have also shown that the increase in PCBs concentration is associated with decreased sympathetic activity up to the second trimester of pregnancy (Lopez-Espinosa et al., 2016). Because of the lack of data, we can’t estimate the PCBs exposure by using two classification variables, which leads to the occurrence of low birth weight infants. In addition, the reasons for the difference in the assessment of PCBs exposure in early and second trimester may be that the stage of some studies is not clear, which leads to subjective consciousness in the process of collecting data. For pregnancy staging, our study was divided into three stages, 1-12 weeks, 13-27 weeks, and 38-42 weeks, respectively. Different countries and regions have different regulations on pregnancy staging, which is one of the effects of the differences.

Our study also showed that PCBs exposure in Europe and America had a more statistically significant impact on birth weight than in Asia. The reason for this problem may be that the differences in analysis and measurement of exposure data are difficult to compare PCB levels from different regions, and different studies use different methods of detection. In the kanae study, the average total TEQ level (WHO,1998) of the mother’s blood was 17.5 TEQ pg / g lipid, which was lower than that of the mother’s plasma TEQ (28.4 TEQ pg / g lipid) in another study in her area (Kanae Konishi, et al.,2009). T. I. et al. Determination of PCBs content by Gas Chromatography and Electron capture. In Marina dengr, the content of PCBs was measured by using an Agilent 7000B gas chromatograph triple quadrupole mass spectrometer (G) (Marina et al., 2014). Although the studies involved are few, can not fully confirm our conclusion, but can provide a direction for future research.

In addition, this meta-analysis has certain limitations. We found high or moderate heterogeneity in most subgroup meta-analysis. These results suggested that heterogeneity in the study may also be affected by other factors, such as economic status, mother’s education, and fetal sex, and We didn’t take into account the limitation of the number of relevant studies in this study. Therefore, further meta-analysis is necessary to explore the source of heterogeneity and more original research in the future.

In Conclusion, the meta analysis revealed a negative correlation between PCBs exposure and birth weight but there was significant difference in the correlation between birth weight loss . Race and exposure assessment items both were the main source of heterogeneity. These results extend our understanding of the adverse effects of PCBs on fetal health and it should be paied more attention for the importance of reducing environmental PCBs pollution and reducing maternal PCBs exposure during pregnancy to improve birth outcomes. More research is needed to further evaluate the health effects of a single PCBs homologue, as well as the effects of different detection methods such as gas chromatography or electron capture.
